# Identification of Novel Alleles Conferring Superior Production of Rose Flavor Phenylethyl Acetate Using Polygenic Analysis in Yeast

**DOI:** 10.1128/mBio.01173-17

**Published:** 2017-11-07

**Authors:** Bruna Trindade de Carvalho, Sylvester Holt, Ben Souffriau, Rogelio Lopes Brandão, Maria R. Foulquié-Moreno, Johan M. Thevelein

**Affiliations:** a Laboratory of Molecular Cell Biology, Institute of Botany and Microbiology, KU Leuven, Flanders, Belgium; b Center for Microbiology, VIB, Leuven-Heverlee, Flanders, Belgium; c Laboratório de Biologia Celular e Molecular, Núcleo de Pesquisas em Ciências Biológicas, ICEB II, Departamento de Farmácia, Escola de Farmácia, Universidade Federal de Ouro Preto, Campus do Morro do Cruzeiro, CEP 35, Ouro Preto, Brazil; Harvard Medical School

**Keywords:** 2-phenylethyl acetate, QTL analysis, fatty acid synthetase, rose flavor, yeast

## Abstract

Flavor compound metabolism is one of the last areas in metabolism where multiple genes encoding biosynthetic enzymes are still unknown. A major challenge is the involvement of side activities of enzymes having their main function in other areas of metabolism. We have applied pooled-segregant whole-genome sequence analysis to identify novel *Saccharomyces cerevisiae* genes affecting production of phenylethyl acetate (2-PEAc). This is a desirable flavor compound of major importance in alcoholic beverages imparting rose- and honey-like aromas, with production of high 2-PEAc levels considered a superior trait. Four quantitative trait loci (QTLs) responsible for high 2-PEAc production were identified, with two loci each showing linkage to the genomes of the BTC.1D and ER18 parents. The first two loci were investigated further. The causative genes were identified by reciprocal allele swapping into both parents using clustered regularly interspaced short palindromic repeat (CRISPR)/Cas9. The superior allele of the first major causative gene, *FAS2*, was dominant and contained two unique single nucleotide polymorphisms (SNPs) responsible for high 2-PEAc production that were not present in other sequenced yeast strains. *FAS2* encodes the alpha subunit of the fatty acid synthetase complex. Surprisingly, the second causative gene was a mutant allele of *TOR1*, a gene involved in nitrogen regulation. Exchange of both superior alleles in the ER18 parent strain increased 2-PEAc production 70%, nearly to the same level as in the best superior segregant. Our results show that polygenic analysis combined with CRISPR/Cas9-mediated allele exchange is a powerful tool for identification of genes encoding missing metabolic enzymes and for development of industrial yeast strains generating novel flavor profiles in alcoholic beverages.

## IMPORTANCE

Multiple reactions in flavor metabolism appear to be catalyzed by side activities of other enzymes that have been difficult to identify. We have applied genetic mapping of quantitative trait loci in the yeast *Saccharomyces cerevisiae* to identify mutant alleles of genes determining the production of phenylethyl acetate, an important flavor compound imparting rose- and honey-like aromas to alcoholic beverages. We identified a unique, dominant allele of *FAS2* that supports high production of phenylethyl acetate. *FAS2* encodes a subunit of the fatty acid synthetase complex and apparently exerts an important side activity on one or more alternative substrates in flavor compound synthesis. The second mutant allele contained a nonsense mutation in *TOR1*, a gene involved in nitrogen regulation of growth. Together the two alleles strongly increased the level of phenylethyl acetate. Our work highlights the potential of genetic mapping of quantitative phenotypic traits to identify novel enzymes and regulatory components in yeast metabolism, including regular metabolic enzymes with unknown side activities responsible for biosynthesis of specific flavor compounds. The superior alleles identified can be used to develop industrial yeast strains generating novel flavor profiles in alcoholic beverages.

## INTRODUCTION

Flavor is one of the main defining characteristics of alcoholic beverages with critical importance for their commercial value (1, 2). The *Saccharomyces cerevisiae* yeast plays an important role in generating the final aroma profile of alcoholic beverages. Pleasant flavors to the human palate include the fruity aromas that are largely derived from secondary metabolism of sugar during yeast alcoholic fermentation. Esters comprise the largest and most important group. They often have pleasant aromas, low perception thresholds, and relatively high concentrations in alcoholic beverages (1, 3, 4). Small changes in ester concentrations can have significant effects on the taste of alcoholic beverages (3).

Esters are formed from an alcohol and a carboxylic acid linked with coenzyme A (CoA). There are two groups of ﬂavor-active esters in fermented beverages, ethyl esters and acetate esters. Ethyl esters are formed from ethanol and the acyl-CoA derivative of medium-chain fatty acids (MCFA). Among this group, the most important are ethyl hexanoate (anise seed, apple-like aroma) and ethyl octanoate (apple, pineapple aroma). Acetate esters are formed from acetyl-CoA and an alcohol that can be ethanol (yielding ethyl acetate) or a higher alcohol derived from amino acid metabolism. The most flavor-relevant acetate esters are ethyl acetate (solvent-like aroma), isoamyl acetate (banana-like aroma), and 2-phenylethylacetate (honey- and rose-like aroma) (1, 4–7).

To date, the main genes responsible for ester biosynthesis identified are *ATF1* and *ATF2* for acetate esters and *EHT1* and *EEB1* for ethyl esters (8–12). Double deletion of *ATF1* and *ATF2* largely abolishes the production of isoamyl acetate and strongly reduces the production of many other flavor esters, including 2-phenylethyl acetate (2-PEAc). The observation that significant production levels are retained in the *atf1Δ atf2Δ* strain indicates involvement of other unknown biosynthetic enzymes (8). Double deletion of *EEB1* and *EHT1* caused considerable but also only partial reduction in the levels of all ethyl esters, again indicating the presence of additional biosynthetic enzymes (10). In addition, little is known about the regulation of the biosynthetic pathways and about other factors that may influence the production of specific flavor compounds. Flavor production is highly variable among yeast strains, and the genetic basis of this broad phenotypic variation has remained largely unknown until recently. Quantitative trait locus (QTL) mapping studies have now revealed genes involved in production of nerolidol, 2-phenyl ethanol, and ethyl esters (13), ethyl acetate (14), and undesirable sulfur flavor compounds (15). Given the large variety of flavor compounds and the many parameters affecting their formation, most of the underlying genetic basis of the natural variation in flavor compound production remains unknown.

Flavor compound production is a complex, polygenic trait. For many years, the genetic analysis of polygenic traits was very cumbersome, but in recent years, powerful tools have been developed that allow efficient mapping of major and minor quantitative trait loci and identification of the causative genes in the center of the QTLs (16–22). These technologies are based on the use of the many single nucleotide polymorphisms (SNPs) between unrelated strains as genetic markers, and the recent development of low-cost high-throughput sequencing technologies has greatly facilitated determination of the nucleotide frequency that is used to detect specific linkage with the genome of one of the parent strains. Pooled-segregant whole-genome sequence analysis has thus emerged as a powerful tool for QTL mapping and has been successfully performed with as few as 20 to 30 superior segregants displaying the phenotype of interest to an extent similar to that of the superior parent strain (17, 21, 23). This low number is important for genetic analysis of nonselectable phenotypes and for traits that require an elaborate experimental setup for scoring, such as a requirement for small-scale fermentations with all individual segregants. The latter has been successfully accomplished for the nonselectable traits of low glycerol production (24, 25), maximum ethanol accumulation capacity (23), ethyl acetate production (14), and production of multiple other flavor compounds (13). Production of flavor compounds, like ethyl acetate, is a nonselectable trait and has to be scored in individual small-scale fermentations with hundreds of segregants to obtain a pool of superior segregants large enough for efficient QTL mapping (13, 14).

In most cases, reciprocal hemizygosity analysis (RHA) has been used for identification of the causative genes in the QTLs (16). It involves construction of two hemizygous diploid strains in which a different allele of the candidate gene has been deleted, followed by comparison of their phenotype for the trait of interest. Bulk RHA has also been used in this bulk RHA; first, consecutive blocks of genes are deleted, followed by deletion of single genes in blocks where a phenotypic difference between the two hemizygous diploid strains is observed in the RHA analysis (22). For production of the flavor compound ethyl acetate, QTL mapping using pooled-segregant whole-genome sequence analysis and RHA for determination of causative genes have resulted in the identification of *PMA1*, *CEM1*, and *TPS1* as novel genes affecting ethyl acetate production (14). The recent development of the clustered regularly interspaced short palindromic repeat (CRISPR)/Cas9 technology for site-directed genetic modification in yeast (26–30) allows us to exchange precisely the causative alleles for their counterparts in the superior and inferior parent, which allows evaluating the contribution of the causative alleles in the haploid parent background.

Polygenic analysis of complex traits has generally been carried out with two haploid parent strains that differ considerably in the trait of interest, i.e., a superior parent and an inferior parent. However, crossing of two strains with similar properties can also generate segregants that strongly differ in these properties and that therefore can be submitted to selection of superior segregants, pooled-segregant whole-genome sequence analysis for QTL mapping and RHA or allele exchange for identification of the causative alleles. Inbreeding populations, in which the genomes of different strains have been mixed to a great extent by multiple meiotic recombinations, have been established for that purpose. This greatly reduces genomic linkage and thus makes the QTLs much smaller, facilitating identification of the causative alleles. This approach has been used for elucidation of the genetic basis of multiple complex traits (13, 31–35). In this case, both parents are bound to harbor causative genetic elements for establishing a superior trait. However, genetic analysis of a complex trait using superior and inferior parents generally also reveals QTLs linked to the inferior parent (17, 20, 22, 23, 36, 37). This indicates the presence of many alleles with potential impact on a specific complex trait as well as the importance of synthetic interactions between appropriate alleles for conferring a complex trait.

In this study, we have investigated the polygenic basis of 2-phenylethyl acetate production, a desirable flavor compound with a honey-, rose-like aroma. Production of higher 2-PEAc levels can thus be considered a superior trait. We have used two random segregants (BTC.1D and ER18) derived from two nonselected diploid strains as parents and identified offspring with high 2-PEAc production. Several major QTLs were then mapped by pooled-segregant whole-genome sequence analysis, and two novel causative alleles of *TOR1* and *FAS2* were identified as superior alleles derived from strain BTC.1D. Exchange of the superior alleles of *TOR1* and *FAS2* by CRISPR/Cas9-mediated allele exchange into the ER18 parent strain established the high 2-PEAc production (70%), indicating that these two superior alleles were responsible for the majority of the high 2-PEAc production capacity. The superior *FAS2* allele contained two unique SNPs, not present in any other sequenced yeast strain, that after exchange into the ER18 parent strain led to a 25% increase in 2-PEAc production. These alleles might be useful tools for construction of cisgenic industrial yeast strains for production of alcoholic beverages with specific modifications of the flavor profile.

## RESULTS

### Flavor profile of a set of segregants obtained from unselected parent strains.

We have obtained a pool of haploid strains for flavor profile analysis by sporulating a hybrid diploid *Saccharomyces cerevisiae* yeast strain, obtained by crossing two descendants from the unrelated industrial yeast strains WLP575, an ale yeast, and Ethanol Red (ER), a bioethanol production yeast. BTC.1D (*MAT***α**), a segregant from WLP575, and ER18 (*MAT***a**), a segregant from ER, were crossed to generate a hybrid diploid named BTC.1D/ER18. This hybrid strain was sporulated, and 574 segregants were isolated and evaluated in small-scale fermentations for flavor production.

**FIG 1:**
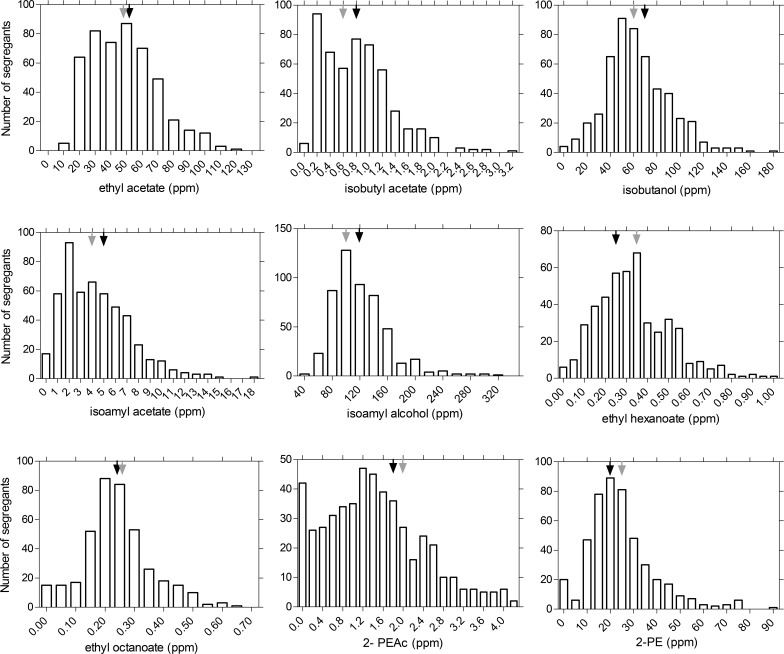
Bar graphs of flavor compound production by the segregants from the BTC.1D/ER18 strain. Fermentations were carried out in 100 ml YP250Glu10%, and flavors were measured by GC-FID at the end of fermentation. The flavor production by the parent strains is represented by black arrows (BTC.1D) and gray arrows (ER18). The lowest value indicated represents either the lowest value measured or the detection limit.

We have used bar graphs to visualize the production data distribution among the segregants for each flavor compound (Fig. 1). Some segregants produced much higher levels of specific flavor compounds than the two parent strains. For isobutyl acetate, one segregant produced 3.14 ppm which is almost 4 times higher than the level produced by the best parent strain (0.85 ppm). Similarly, the highest production of ethyl acetate reached in the population of segregants was 123 ppm, whereas the parent strains produced 53.6 and 47.2 ppm. For 2-PEAc, one of the segregants produced 4 ppm, while the BTC.1D and ER18 parent strains produced only 1.7 and 1.9 ppm, respectively. Because 2-PEAc is an important and highly desirable ester in alcoholic beverages due to its pleasant rose-like and honey-like aroma and since little is known about factors controlling its synthesis, we have selected this flavor production trait for genetic analysis.

### Pooled-segregant whole-genome sequence analysis and QTL mapping.

We have composed a pool of 24 selected, superior segregants with a 2-PEAc production level of at least 2.8 ppm, corresponding to the 2-PEAc level produced by the 98th percentile of the MCB yeast strain collection, and a second “unselected pool” with 24 random segregants. Both pools were subjected to pooled-segregant whole-genome sequence analysis using Illumina HiSeq2000 technology (Beijing Genomics Institute [BGI], Hong Kong, China).

**FIG 2:**
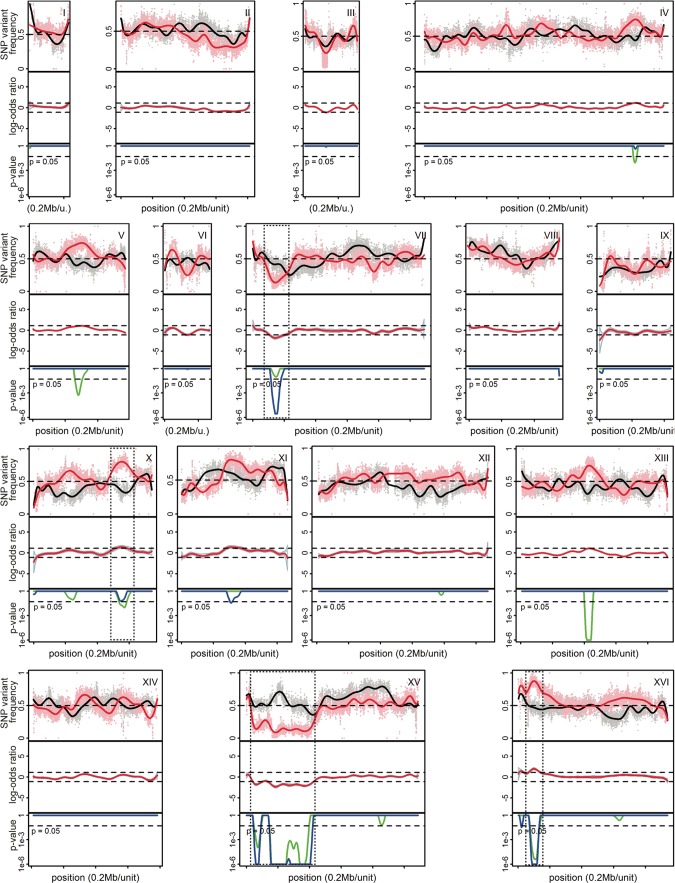
Genetic mapping of QTLs involved in high phenylethyl acetate production by pooled-segregant whole-genome sequence analysis. Pooled F1 selected or random segregants (24 segregants in each pool) were subjected to sequence analysis with the Illumina platform at BGI. The chromosome number is shown in the top right corner of each set of graphs. The SNP variant frequency was used for QTL mapping. Black lines represent the random “unselected pool,” and red lines represent the selected “superior pool” (top rows). The log odds ratio with confidence interval is shown in the second rows. The most reliable QTLs are indicated by stippled rectangles. When the SNP variant frequency is >0.5, linkage is with the BTC.1D parent. When the SNP variant frequency is <0.5, linkage is with the ER18 parent. *P* values calculated from the sequencing data were plotted against the respective chromosomal position (third rows). *P* values of <0.05 (indicated by dotted line) were considered statistically significant. The blue line represents *P* values calculated with 0.5 as reference for the whole genome. The green line represents *P* values calculated with the actual result of the random pool as the reference for the whole genome.

The sequence reads from the parental strains and the pool were first aligned to the reference S288c genome sequence to identify all SNPs after which the SNPs between the two parent strains with a coverage of at least 20 times were selected following previous polygenic analysis protocols (17, 38). SNPs of the individual parent strains with the S288c reference genome sequence but absent in the parent strains themselves (i.e., the same nucleotide was present in the two parent strains) were filtered out using the next-generation sequencing eclipse plugin (NGSEP) program. The SNP variant frequency was used for QTL mapping using the NGSEP program in order to identify the genomic loci linked to high 2-PEAc production. For this purpose, the SNP variant frequency was plotted against the chromosomal position of the SNP using version 0.99.903 of the RStudio program, generating the QTL map (Fig. 2). Next, *P* values calculated from the SNP variant frequency data were plotted against the respective chromosomal position. *P* values of <0.05 were considered statistically significant. Because of the low number of segregants and the unexpectedly large variation of the SNP variant frequency in the random pool, we calculated the *P* values for the whole genome both with the mapping result of the random pool as a reference and also with the mean value of 0.5 for the whole genome as a reference, which is the value that would normally be expected for most of the genome (23) (Fig. 2).

Since genomic elements of both parent strains could contribute to the high 2-PEAc phenotype in the superior pool, the QTLs could be linked to either parent genome. A high SNP frequency, significantly above 50%, indicated linkage with the corresponding part of the genome of the BTC.1D parent strain, while a low SNP frequency indicated linkage with the corresponding part of the genome of the ER18 parent strain. The midline obtained fluctuated around 50% inheritance in most areas of the genome. Strong deviation from 50% inheritance was observed on chromosomes VII, X, XV, and XVI. The QTLs on chromosomes X and XVI were linked to the BTC.1D parent genome, while the QTLs on chromosomes VII and XV were linked to the ER18 parent genome (Fig. 2). We have selected the QTLs on chromosomes X and XVI linked to the BTC.1D parent for further analysis.

### Identification of *TOR1* as a causative gene in the major QTL on chromosome X.

**FIG 3:**
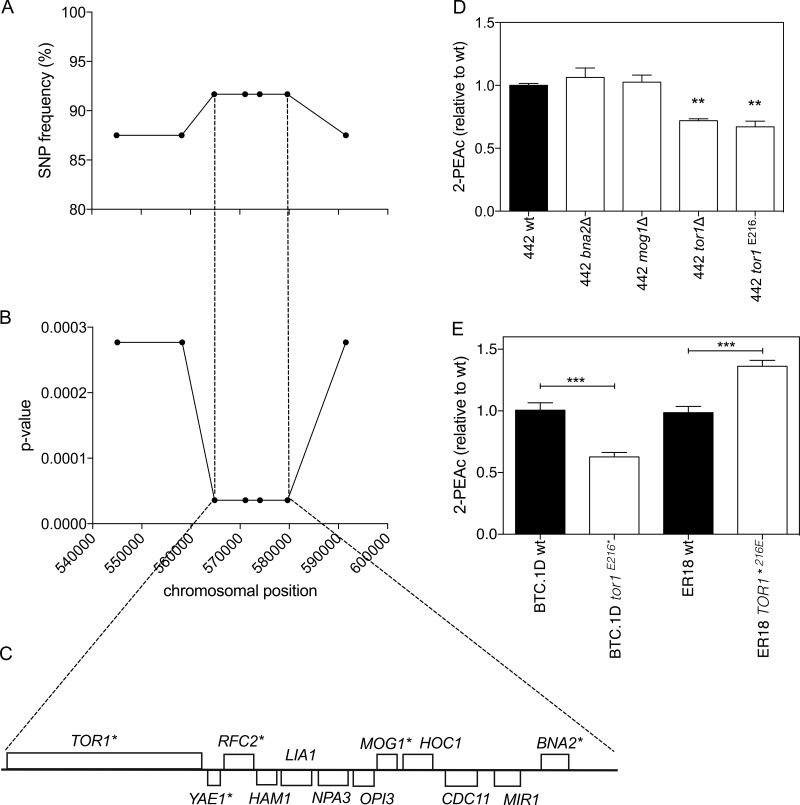
Identification of *TOR1* as a causative gene in QTL1 located on chromosome X. (A) SNP variant frequency for seven selected SNPs as determined with the 24 superior segregants individually. (B) *P* values for the same seven SNPs plotted against their chromosomal position. *P* values below 0.05 were considered statistically significant. (C) Overview of all genes present in the region with strongest linkage of the QTL1 on chromosome X. Genes marked with an asterisk contain one or more nonsynonymous mutations in the ORF. (D) 2-PEAc production in strains derived from superior segregant 442: wild-type (wt), *tor1*Δ, and *tor1*^E216^* strains. Fermentations were carried out in 100 ml YP250Glu10%. 2-PEAc was measured at the end of fermentation. Values that were significantly different (*P* < 0.01) by the unpaired Student *t* test from the value for 2-PEAc production for the wild-type strain are indicated by two asterisks. (E) *TOR1* allele swapping in the parent strains BTC.1D and ER18. Fermentations were carried out in 100 ml YP250Glu10%. 2-PEAc was measured at the end of fermentation. Values for 2-PEAc production that were significantly different (*P* < 0.001) by the unpaired Student *t* test are indicated by a bar and three asterisks.

First, we have fine mapped the major QTL on chromosome X by scoring selected SNPs by allele-specific PCR in the 24 individual superior segregants. Random SNPs were chosen throughout the QTL region at an average distance of 5 to 15 kb, resulting in a total of seven SNPs. The whole region of 46.5 kb showed strong linkage, with a *P* value lower than 0.001. The highest linkage (*P* value of 3.6 × 10^−5^) was observed for a 15-kb region located in the middle of the QTL. This region contained 12 genes, of which 5 had nonsynonymous mutations in the open reading frame (ORF) (Fig. 3A to C). Among these five genes, three were nonessential genes and their possible effect on the 2-PEAc phenotype was verified by deleting each gene individually.

Since neither the haploid parents nor the diploid hybrid showed the superior phenotype, the possible involvement of the candidate genes was investigated in a superior segregant, strain 442. This haploid strain exhibited a 2-PEAc production of about 4 ppm and contained the superior allele in the region under investigation. The nonessential genes *MOG1*, *BNA2*, and *TOR1* were deleted in this superior segregant by replacing them with a nourseothricin resistance marker cassette. The deletion strains were evaluated by fermentation for flavor production, and a significant decrease in 2-PEAc production was observed only for the 442 *tor1*Δ strain (Fig. 3D).

Sequence comparison of the *TOR1* alleles from the two parents revealed several polymorphisms, eight of them leading to nonsynonymous mutations (Table 1). The most striking mutation was an early stop codon at position 216 of the amino acid sequence in the ER18 parent, while the wild-type Tor1 protein has a total length of 2,470 amino acids. The truncated protein was expected to cause a phenotype similar to that of the 442 *tor1*Δ strain. We inserted this specific point mutation in the superior segregant 442 by CRISPR/Cas9 methodology and compared the phenotype of the mutant strain with that of the wild-type 442 and 442 *tor1*Δ strains. Figure 3D shows that the 442 *tor1*Δ and 442 *tor1*^E216^* strains produced similarly reduced levels of 2-PEAc, which were approximately 30% lower than that of the wild-type strain. This indicated that *TOR1* affected 2-PEAc production and that the *tor1*^E216^* allele, derived from strain ER18, behaved as a loss-of-function allele.

**TABLE 1:** Occurrence of nonsynonymous mutations in the proteins Tor1 and Fas2 in a set of 39 yeast strains for which the complete genome sequence is known

Strain	Amino acid in Tor1 at position:								Amino acid in Fas2 at position:				
	58	133	216	396	547	1117	1640	2414	57	565	1136	1624	1800
BTC.1D^a^	**D**	**S**	**E**	**N**	**N**	**S**	**F**	**K**	**T**	**N**	**T**	**I**	**N**
ER18^a^	**G**	**N**	*	**K**	**S**	**P**	**V**	**R**	**A**	**S**	**A**	**V**	**S**
S288c	D	S	E	N	N	S	F	K	A	S	A	V	S
AWRI796		P	F	T	Y			K		N	A	V	N
BC 187	D	S	E	K	S	S	F	K	T	N	A	V	N
BY4741	D	S	E	N	N	S	F	K	A	S	A	V	S
BY4742	D	S	E	N	N	S	F	K	A	S	A	V	S
CBS7960			E	K	S	S	V	R	A	S	A	V	S
CEN.PK	D	S	E	K	S	S	V	R	A	S	A	V	N
D273-10B	G	N	E	K	S	S	V	R	A	S	A	V	N
DBVP6044	G	N	E	K	S	S	V	R	A	N	A	V	N
EC1118	D	S	E	K	S	S	V	K	A	N	A	V	N
EC9-8	G	N	E	K	S	S			T	N	A	V	N
FL100	D	S	E	N	N	S	F	K	A	S	A	V	S
FY1679	D	S	E	N	N	S	F	K	X	N	A	V	N
FostersB	P	P	F	T	Y			X	A	N	A	V	N
FostersO			E	K	S				A	N	A	V	N
JAY291	D	S	E	K	S	S	V	R	A	S	A	V	S
JK9-3d	D	S	E	K	S	S	V	R	A	S	A	V	S
K11	G	N	E	K	S	P	V	R	A	S	A	V	S
Kyokai7	G	N	E	K	S	P	V	R	T	N	A	V	N
L1528	D	S	E	K	S	S	F	K	A	N	A	V	N
LalvinQA23					Y				A	S	A	V	N
RM11-1a	D	S	E	K	S	S	F	K	T	N	A	V	N
RedStar	G	N	E	K	S	S	F	R	T	N	A	V	N
SEY6210	D	S	E	K	S	S	V	R	A	S	A	V	S
SK1	G	N	E	K	S	S	V	R	A	S	A	V	N
UC5	G	N	E	K	S	P	V	R	A	S	A	V	S
VL3	D	S	E	K	S	S			A	S	A	V	S
Vin13	D	S	E	K	S				A	S	A	V	S
W303	D	S	E	N	N	S	F	K	T	N	A	V	N
X2180-1A	D	S	E	N	N	S	F	K	X	N	A		
Y55	G	N	E	K	S	S	V	R	A	S	A	V	S
YJM269	G	N	E	K	S	S	V	R	A	S	A	V	S
YJM339	G	N	E	K	S	P	V	R	T	N	A	V	N
YJM789	G	N	E	K	S	P	V	R	A	S	A	V	S
YJM499	D	S	E	K	S	S	V	R	T	N	A	V	N
YPS128	G	N	E	K	S	S	V	R	A	S	A	V	S
YPS163	G	N	E	K	S	S	V	R	A	S	A	V	N
YS9	P	P	F	T	Y			R	A	S	A	V	N
ZTW1	G	N	E	K	S	S	V	R	T	N	A	V	N

^a^ The amino acids for parental strains BTC.1D and ER18 are shown in boldface type. The asterisk indicates a nonsense mutation.

The *TOR1* alleles were also exchanged in the parent strains. The stop codon mutation E216* was introduced in the BTC.1D parent, while the E216 residue was restored in the ER18 parent, both via CRISPR/Cas9 methodology. The 2-PEAc production by the BTC.1D tor1^E216^* and ER18 *TOR1*^E216^ strains was evaluated in small-scale fermentations. The introduction of the early stop codon into the BTC.1D parent resulted in a decrease of about 40% in 2-PEAc production, while ER18 showed a 35% increase when the E216 residue was restored and the complete wild-type Tor protein was therefore expressed (Fig. 3E). These results further confirmed that *TOR1* was the causative gene in the major QTL on chromosome X and that Tor1 inactivation compromises 2-PEAc production.

### Identification of *FAS2* as a causative gene in the major QTL on chromosome XVI.

**FIG 4:**
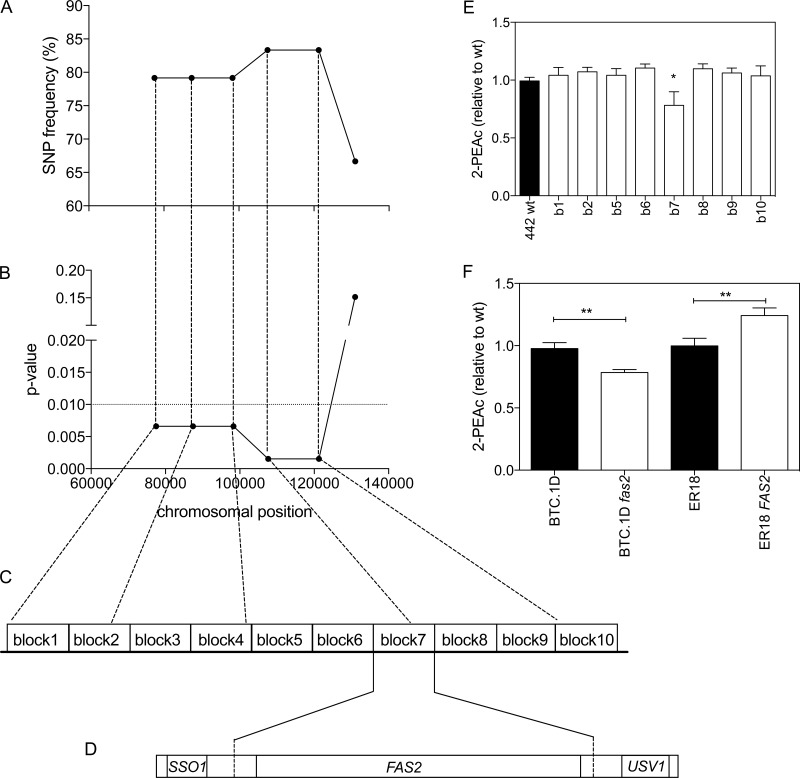
Identification of *FAS2* as a causative gene in QTL2 located on chromosome XVI. (A) SNP variant frequency for six selected SNPs as determined with the 24 superior segregants individually. (B) *P* values for the same six SNPs. *P* values below 0.05 were considered statistically significant. (C) QTL2 was divided into 10 gene blocks, which were each tested for causative character using CRISPR/Cas9 direct replacement by the 2xgRNA approach. (D) Block 7 contained the *FAS2* gene, which turned out to be causative for QTL2. (E) 2-PEAc production by the wild-type (wt) strain 442 and derivatives with a bulk replacement of each gene block (gene block 1 [b1] to 10 [b10]). Fermentations were carried out in 100 ml YP250Glu10%. 2-PEAc was measured at the end of fermentation. The value for 2-PEAc production was significantly different (*P* < 0.05) from the value for the wild type by the unpaired Student *t* test as indicated by the asterisk. (F) *FAS2* allele swapping in the parent strains BTC.1D and ER18. Fermentations were carried out in 100 ml YP250Glu10%. 2-PEAc was measured at the end of fermentation. The 2-PEAc production values indicated by a bar and two asterisks were significantly different (*P* < 0.01) by the unpaired Student *t* test.

We have performed fine mapping with allele-specific PCR to confirm and downscale the major QTL on chromosome XVI. Six SNPs were selected; these SNPs covered a region of 54 kb. The region between the first and fifth SNP showed strong linkage with the BTC.1D parent with *P* values ranging from 0.002 to 0.007 (Fig. 4A and B). This area consists of 25 genes, most of them containing nonsynonymous mutations in the ORF as well as mutations in the promoter and/or terminator regions. The region was divided in 10 blocks in order to evaluate consecutive sets of genes for location of the causative gene in a process of bulk replacement (Fig. 4C). As for the major QTL on chromosome X, we chose the superior segregant 442, which contained the genomic sequence of the BTC.1D parent over the whole length of this QTL, for exchange of the selected blocks of genes.

Direct replacement of the blocks of genes was required, since the superior segregant 442 is haploid and some genes in the blocks are essential. Direct replacement was achieved using CRISPR/Cas9 methodology using two guide RNAs (gRNAs), one targeting a sequence upstream of the first SNP of interest and the other targeting a sequence downstream of the last SNP of interest. The donor DNA was PCR amplified from the ER18 parent, which apparently contained the genomic DNA with the inferior causative allele. The bulk replacement strains for each block of genes were used in small-scale fermentations and 2-PEAc production was measured at the end of fermentation. This revealed a conspicuous drop in 2-PEAc production specifically with the 442 strain in which block number 7 with the BTC.1D parent sequence had been exchanged for the sequence of the ER18 parent strain (Fig. 4E).

The length of block 7 was 6.4 kb, and it contained one gene (*FAS2*) with about 500 bp of its promoter and terminator. Since *FAS2* encodes the alpha subunit of fatty acid synthetase, it appeared to be the best candidate causative gene in this block (Fig. 4D). Comparison of the *FAS2* gene sequences of the BTC.1D and ER18 parent strains revealed five nonsynonymous mutations in the open reading frame. We checked the occurrence of those amino acid mutations in 39 strains for which the whole-genome sequence is available in the *Saccharomyces* Genome Database. The results are shown in Table 1. Fas2 is a highly conserved protein, and of the five amino acid changes found in the BTC.1D parent sequence, two were not found in any sequenced strain. At position 1136 in the amino acid sequence, strain BTC.1D contained threonine, while all the other strains contained alanine, while at position 1624 in the amino acid sequence, BTC.1D contained isoleucine, while it was valine in all other strains. For practical reasons, the BTC.1D superior allele, *FAS2*^A57T,S565N,A1136T,V1624I,S1800N^, will be referred to as *FAS2*^BTC.1D^ and the inferior allele from strain ER18 will be referred to as *fas2*^ER18^.

In order to confirm *FAS2* as the causative allele in the major QTL on chromosome XVI, we performed *FAS2* allele swapping in the parent strains by targeting the same cutting sites used to construct the bulk replacement in segregant 442. The production of 2-PEAc by the newly constructed transformant strains was compared with that of the wild-type parents BTC.1D and ER18. Figure 4F shows that the replacement of *FAS2*^BTC.1D^ by the *fas2*^ER18^ allele in the BTC.1D parent led to a 25% reduction in 2-PEAc production, while the replacement of *fas2*^ER18^ by the *FAS2*^BTC.1D^ allele in the ER18 parent raised it by 30%. We did not test the effect of *FAS2* deletion on 2-PEAc production because *FAS2* is an essential gene.

### Establishing the superior phenotype by replacing the *TOR1* and *FAS2* alleles in the ER18 parent strain.

**FIG 5:**
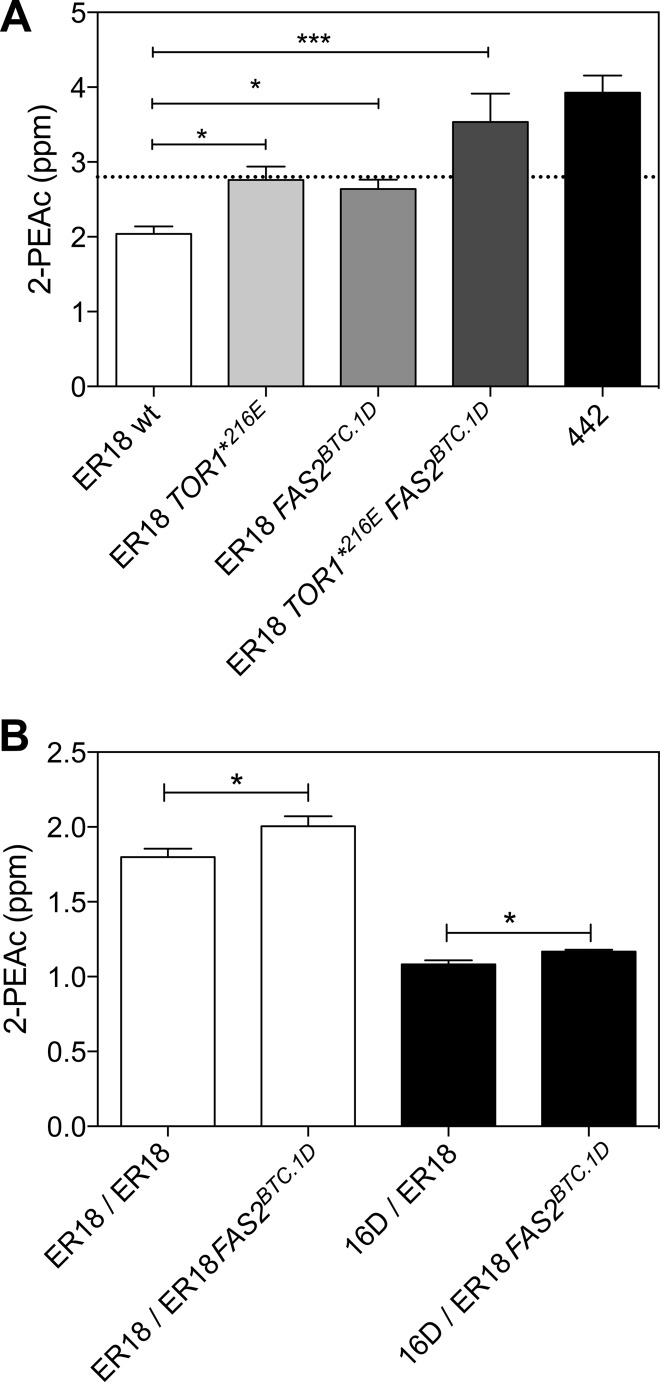
Allele swapping of both causative alleles from strain BTC-1D into the ER18 parent strain and test for dominance/recessivity of the *FAS2*^BTC.1D^ allele. (A) The *tor1*^E216^* SNP in parent strain ER18 was changed into the wild-type *TOR1*^E216^ amino acid from parent strain BTC.1D, and the *FAS2*^ER18^ allele in parent strain ER18 was replaced by the *FAS2*^BTC.1D^ allele from parent strain BTC.1D, while in the third ER18-derived strain, both changes were introduced. 2-PEAc production by the parent strain ER18, the single and double replacement strains, and strain 442, one of the top 2-PEAc-producing segregants, is shown. Fermentations were carried out in 100 ml YP250Glu10%. 2-PEAc was measured at the end of fermentation. The values for 2-PEAc production were significantly different by one-way analysis of variance (ANOVA) with Dunnet’s multiple-comparison test as indicated by a bar and asterisks as follows: *, *P* < 0.05; ***, *P* < 0.001. (B) The strains ER18 TOR1*^216E^ and ER18 TOR1*^216E^ *FAS2*^BTC.1D^ were crossed with ER18 TOR1*^216E^ (white bars) and 16D (black bars). Fermentations were carried out in 100 ml YP250Glu10%. 2-PEAc was measured at the end of fermentation. The values for 2-PEAc production were significantly different (*P* < 0.05) by the unpaired *t* test as indicated by the bar and single asterisk.

QTL mapping revealed four major QTLs linked to high production of 2-PEAc. Allele-specific PCR analysis with the two parent strains confirmed that the ER18 parent contained the superior allele for the QTLs on chromosomes VII and XV and the inferior allele for the QTLs on chromosomes X and XVI, in which we identified *TOR1* and *FAS2*, respectively, as the causative genes. Hence, we decided to replace both the inferior alleles *tor1*^E216^* and *fas*2^ER18^ by the *TOR1*^E216^ and *FAS2*^BTC.1D^ superior alleles, respectively, in the ER18 parent strain to obtain a strain possessing superior alleles of the four major QTLs (Fig. 5A). Also the single replacement strains were constructed. The original ER18 parent strain and the modified ER18 strains were used in small-scale fermentations, and 2-PEAc production was analyzed. This showed that the replacement of *tor1*^E216^* was already enough to reach the superior phenotype of the selected segregants (≥2.8 ppm 2-PEAc), while the replacement of both inferior alleles, *tor1*^E216^* and *fas2*^ER18^, led to an increase of about 70% in 2-PEAc production, reaching about 3.5 ppm, almost as high as the level of 4 ppm for the top superior segregant 442 (Fig. 5A).

To test whether the *FAS2*^*BTC.1D*^ allele was dominant or recessive in the same genetic background, we crossed the control strain ER18 (*MAT*α) and the strain ER18 FAS2^BTC.1D^ (*MAT*α) with strain ER18 (*MAT***a**) to obtain the two diploid strains ER18/ER18 and ER18/ER18 FAS2^BTC.1D^ (Fig. 5B). To perform the same test with a different genetic background, we crossed the control strain ER18 (*MAT*α) and the strain ER18 FAS2^BTC.1D^ (*MAT*α) with strain 16D (*MAT***a**) to obtain the diploid strains ER18/16D and ER18 FAS2^BTC^.1D/16D (Fig. 5B). Strain 16D is a haploid segregant of JT22689, a strain used in “sturm” must fermentations (Austria) (21), which contains the *fas2*^*ER18*^ allele. In all of the constructed diploid strains, any mutant *TOR1* allele with the early stop codon E216* was corrected into the wild-type *TOR1* allele. Therefore, any difference between the diploid strain pairs would be due only to the effect of the *FAS2*^*BTC.1D*^ allele. (The *TOR1* gene was therefore not mentioned in the genotypes indicated in Fig. 5B.) In both diploid strain pairs, the presence of a wild-type *FAS2* copy together with one copy of the superior allele *FAS2*^*BTC.1D*^ in the same strain was sufficient to increase 2-PEAc production significantly above that in the control strain containing two *FAS2* wild-type alleles, indicating the dominant character of the *FAS2*^*BTC.1D*^ allele (Fig. 5B).

### Production of the other flavor compounds in the ER18 strains engineered for high 2-PEAc production.

**FIG 6:**
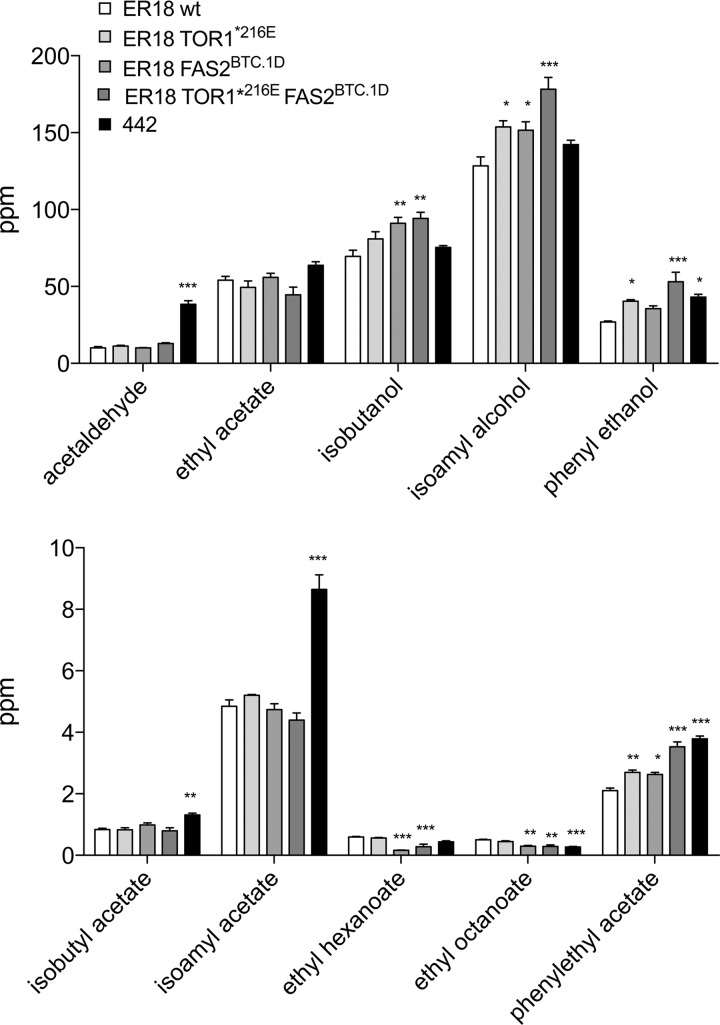
Evaluation of the complete flavor profile of the engineered ER18 strains. The *tor1*^E216^* SNP in parent strain ER18 was changed into the wild-type *TOR1*^E216^ amino acid from parent strain BTC.1D, and the *FAS2*^ER18^ allele in parent strain ER18 was replaced by the *FAS2*^BTC.1D^ allele from parent strain BTC.1D, while in the third ER18-derived strain, both changes were introduced. The production of different flavor compounds by the wild-type parent strain ER18, the single replacement strains (ER18 *TOR1*^*E216*^ and ER18 *FAS2*^*BTC.1D*^), double replacement strains, and strain 442, one of the top 2-PEAc-producing segregants, is shown. Fermentations were carried out in 100 ml YP250Glu10% at 140 rpm and 25°C. Flavor compounds were measured at the end of fermentation. For each flavor compound, the one-way ANOVA with Dunnet’s multiple-comparison test was applied to determine significance of any difference with the control strain ER18 (*, *P* < 0.05; **, *P* < 0.01; ***, *P* < 0.001).

The single and double replacement ER18 strains were also evaluated for the production of other flavor compounds in YP250Glu10% (0.27% yeast extract, 0.54% Bacto peptone to a total predicted nitrogen content of 250 mg/liter, which is in the same range as in beer and wine fermentations, and 10% [wt/vol] glucose) medium (Fig. 6). For none of the other acetate esters, ethyl, isobutyl, and isoamyl acetate, was there any significant change in the production level compared to the parent ER18 strain. Hence, in YP250Glu10% medium, the mutations causing the increase in 2-PEAc production do not have a general effect on acetate ester production. On the other hand, for isobutanol, isoamyl alcohol, and particularly phenylethanol, there appeared to be an increase similar to that observed for phenylethyl acetate. The increase in phenylethanol, which also has a rose flavor, may strengthen the effect of the enhanced 2-PEAc level on total rose flavor impression. The higher increase in phenylethanol (97%) compared to isobutanol and isoamyl alcohol (35% and 39%, respectively) may explain why only the increase in phenylethyl acetate was significant and not that in isobutyl acetate and isoamyl acetate. The *FAS2* allele caused a strong reduction in the production of ethyl hexanoate and ethyl octanoate, which may be due to reduced activity of Fas2 for synthesis of medium-chain fatty acids and/or indicate competition between the synthesis of these compounds and the synthesis of 2-PEAc. For acetaldehyde, there was no significant difference among the four strains.

**FIG 7:**
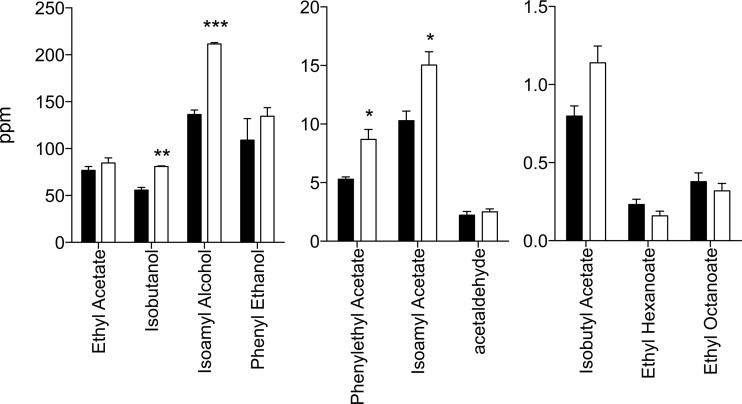
Evaluation of the flavor profile in brewing wort of strain ER18 and its *TOR1 FAS2* double replacement strain. The TOR1*^216E^ and *FAS2*^BTC.1D^ alleles were engineered into the ER18 strain to obtain the ER18TOR1*^216E^ *FAS2*^BTC.1D^ strain. Both strains were used for fermentations in 17°P Brouwland brewing wort with 20 ppm O_2_ at 20°C. Flavor compounds were measured at the end of fermentation. For each flavor compound, the unpaired *t* test was applied to determine significance of any difference between the two strains (*, *P* < 0.05; **, *P* < 0.01; ***, *P* < 0.001).

The double replacement strain, containing *TOR1*^E216^ and *FAS2*^BTC.1D^, was also compared with the parent strain in wort fermentations (Fig. 7). This revealed a 60% increase in the level of 2-PEAc, confirming the potency of the superior alleles to enhance rose flavor production. As in YP medium, the level of isobutanol and isoamyl alcohol, but not that of phenyl ethanol, was significantly increased. The double replacement strain also showed a significant increase in both isoamyl alcohol and isoamyl acetate, leaving the ratio unchanged, as opposed to YP medium where only isoamyl alcohol increased and not isoamyl acetate. Interestingly, in wort medium, there was no significant drop in the production of ethyl hexanoate and ethyl octanoate, as opposed to YP medium.

## DISCUSSION

Polygenic analysis is turning out to be a powerful tool to identify novel components involved in physiological and biochemical traits by addressing natural variation in an unbiased way. Examples include *MKT1* in sporulation capacity (39), high-temperature growth (40), and mitochondrial genome stability (41), and global gene expression (42), *APJ1* in ethanol tolerance (17), *KIN3* in maximal ethanol accumulation capacity (23), *CUP2*, *GLO1*, and *DOT5* in acetic acid tolerance (21), *NCS2*, *PRP42*, and *SMD2* in thermotolerance (22, 43) and also for wine aroma production with *PDR8* in the production of farnesyldiphosphate derivatives, *ABZ1* in the production of numerous flavor compounds, *PLB2* in ethyl ester synthesis (13), and *IRC7* in production of the varietal thiol 4-mercapto-4-methylpentan-2-one (4MMP) (44).

In this work, we have used segregants from nonselected parent strains for phenotyping and QTL mapping by pooled-segregant whole-genome sequence analysis. The production of 2-PEAc in the segregants ranged from less than 0.1 ppm to about 4.0 ppm, and many segregants produced a higher level of 2-PEAc than the parent strains, which produced about 2 ppm (1.74 and 1.9 ppm). Steyer et al. (13) reported that segregants from a cross between a laboratory strain and a wine strain could exceed the production level of both parents for some flavor compounds, which was most pronounced for acetate esters. The flavor threshold values reported for 2-PEAc in wine and beer differ greatly, ranging from 0.25 (in the presence of 10% ethanol) to 3.8 ppm (2, 7, 45). We decided to take a stringent cutoff of at least 2.8 ppm for 2-PEAc production by the segregants. This corresponds to a very high level of 2-PEAc production, present in only a small minority of natural and industrial yeast strains. It resulted in a pool of 24 superior segregants.

In this work, we have identified two new genetic elements, *FAS2* and *TOR1*, that have never previously been connected to production of 2-PEAc, an important flavor compound in alcoholic beverages, imparting a honey- and rose-like flavor. Because of the severe truncation, the *tor1*^E216^* allele likely produces an inactive gene product. While the identification of *TOR1* as a causative allele reveals an unexpected link between TOR signaling and 2-PEAc production, the molecular connection remains unclear.

*TOR1* encodes a phosphatidylinositol kinase homologue that regulates multiple cellular processes, including induction of autophagy (46) and the diauxic shift, and it directly modulates glucose activation and discrimination pathways (47). Tor1 has never been directly associated with flavor production, although its major downstream target Sch9 has been found as an important regulator for *ATF1* expression (48). Tor1 is well-known to be involved in nitrogen-regulated processes, such as nitrogen catabolite repression (49, 50). Since most industrial fermentation processes with the yeast *Saccharomyces cerevisiae* happen under nitrogen starvation and since flavor production is strongly affected by nitrogen availability (51), the defective *TOR1* allele identified in our study may affect 2-PEAc production through interference with nitrogen metabolism. Tor signaling also regulates the transcription of amino acid permeases and therefore might influence the levels of l-phenylalanine and its breakdown product 2-phenylethanol (2-PE) as precursors of 2-PEAc synthesis (52). Insertion of the nonsense mutant allele *tor1*^E216^* in the superior segregant 442 and the BTC.1D parent strain led to a decrease of about 30% in 2-PEAc production. Fermentation performance was not affected by this *TOR1* replacement or by its deletion, and therefore, the reduction in ester production is not due to sluggish or incomplete fermentation. This nonsense mutation was not present in any other *S. cerevisiae* strain in which the genome has been completely sequenced. The usefulness of this defective allele for industrial strain improvement therefore appears very limited, since most natural and industrial yeast strains likely also have an intact wild-type *TOR1* allele.

**FIG 8:**
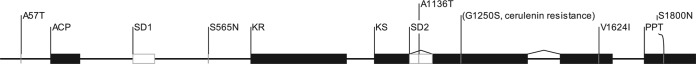
Locations of the mutant amino acid residues identified in the Fas2 alpha subunit of the fatty acid synthase complex. The huge Fas2 protein contains domains for acyl carrier protein (ACP), ketoacyl reductase (KR), ketoacyl synthase (KS), phosphopantetheinyl transferase (PPT), and two structural domains (SD1 and SD2) (87). The mutations identified in this paper, A57T, S565N, A1136T, V1624I, and S1800N, are indicated. A1136T and V1624I are unique among sequenced *S. cerevisiae* strains. Also indicated is the previously identified G1250S mutation that causes both increased production of ethyl hexanoate and cerulenin resistance.

On the other hand, the *FAS2* gene, which encodes the alpha subunit of fatty acid synthetase may well be a genuine enzyme of 2-PEAc metabolism. It encodes a very large, multifunctional protein of 1,887 amino acids (aa), which contributes acyl-carrier, 3-ketoreductase, 3-ketosynthase, and phosphopantetheinyl transferase activities to the fatty acid synthetase complex (53) (Fig. 8). The latter is composed of six Fas1 and six Fas2 subunits. It is known that the microbial type I fatty acid synthases (FASs) are involved in multiple functions linked to fatty acid synthesis: *de novo* synthesis of long-chain fatty acids, mitochondrial fatty acid synthesis, acylation of certain secondary metabolites and coenzymes, fatty acid elongation, and synthesis of the vast diversity of mycobacterial lipids (54). FAS activity has previously not been connected to production of 2-PEAc or other acetate esters. The mutations in the *FAS2*^A57T,S565N,A1136T,V1624I,S1800N^ allele are located upstream of the acyl-carrier domain in the N terminus (A57T), upstream of the 3-ketoreductase domain (S565N), in the structural subdomain SD2 (A1136T) of the 3-ketosynthase domain which also contains a second mutation in the downstream portion (V1624I) and in the phosphopantetheinyl transferase domain (S1800N). Hence, there are two mutations in known functional domains but how these could influence 2-PEAc production is not clear. The mutations in Fas2 may compromise acetyl-CoA utilization by the fatty acid synthase complex and in this way result in modified acetyl-CoA homeostasis in the cell, in turn causing higher availability of acetyl-CoA as the substrate for 2-PEAc synthesis by the *ATF1-* and *ATF2*-encoded alcohol acetyl-CoA transferases. The *FAS2*^BTC.1D^ allele might thus act as a dominant-negative allele on fatty acid biosynthesis.

Previous work has established a connection between flavor ester production in yeast and the *FAS2* gene product. This work was based on the finding that mutant yeast strains resistant to cerulenin contained a dominant mutation, Gly1250Ser, in *FAS2* and at the same time showed higher production levels of ethyl caproate, a compound producing highly desirable apple-like flavors in sake (55, 56). The cerulenin resistance conferring mutation G1250S is located in the middle of the 3-ketosynthase domain (Fig. 8). Much work in this respect has been performed for the construction of self-cloned nongenetically modified organism (non-GMO) sake strains with mutations in *FAS2* that enhance ethyl caproate production and were obtained on the basis of cerulenin resistance (57–62). There is little information, however, concerning the possible effect of these cerulenin resistance-conferring mutations in *FAS2* on the production of other flavor compounds in yeast. This approach to improve the flavor profile of an alcoholic beverage has been very limited due to the very specific nature of the cerulenin resistance-conferring mutations. Our work using polygenic analysis of flavor compound production allows for a much broader and unbiased approach to identify novel genes involved, directly or indirectly, in the production of specific flavor compounds.

In the present study, we have identified a naturally occurring *FAS2* allele conferring superior production of 2-PEAc, but other flavor compounds were also affected. The level of phenylethanol was also significantly increased but only in synthetic medium. If this could also happen in wort medium under other conditions, it would strengthen the increase in perception of rose flavor in beer. Another positive effect on flavor production is that all acetate esters are enhanced except for ethyl acetate, which has a generally negative solvent-like off-flavor. On the other hand, there was a conspicuous drop in the positive-flavor compounds ethyl hexanoate and ethyl octanoate, but this was observed only in synthetic medium and not in wort medium. There was also an increase in the level of the higher alcohols, isoamyl alcohol and isobutanol, which may have a positive or negative effect on flavor perception. This depends on the general flavor profile produced by the strain genetic background, in particular the total level of higher alcohols already produced by the strain, as well as the application of high-gravity brewing, which generally causes more production of higher alcohols (63).

This superior *FAS2*^BTC.1D^ allele does not have the mutation causing the G1250S amino acid change that provides cerulenin resistance. On the other hand, it contains other SNPs causing five amino acid changes compared to *fas2*^ER18^, three of which are also found in other yeast strains. The mutations A1136T and V1624I are not found in any other sequenced *S. cerevisiae* strain and might therefore be unique mutations responsible for the superior acetate ester aroma profile brought about by the BTC.1D allele. Our work has now revealed that the *FAS2* gene product may play a much more important role in the synthesis of flavor esters than previously anticipated, since not only 2-PEAc production was affected by the *FAS2*^BTC.1D^ allele but the production of other flavor compounds as well. The *FAS2* mutation may compromise the cycling of the fatty acids within the fatty acid synthetase complex until they reach the appropriate length. Precocious release of hexanoyl-CoA from the complex may explain why the level of ethyl hexanoate was also strongly enhanced. The mutations in the fatty acid synthetase complex may disturb lipid homeostasis, causing effects on the production of multiple metabolically connected compounds, like the flavor esters.

2-PEAc is an acetate ester made by esterification of acetyl-CoA and 2-phenylethanol. 2-PE is synthesized by degradation of the aromatic amino acid l-phenylalanine. Increased levels of 2-PEAc can be due to higher levels of its precursor, 2-PE. Indeed, mutants of Kyokai no. 9, a sake yeast strain, that were selected for higher 2-PE production also showed higher levels of 2-PEAc (64). Mutants with enhanced 2-PE production were obtained by chemical mutagenesis, followed by selection for *p*-fluoro-DL-phenylalanine (PFP) resistance. PFP is a phenylalanine analog, and PFP-resistant mutants are usually deficient in phenylalanine feedback inhibition of *ARO3*-encoded 3-deoxy-d-arabino-heptulosonate-7-phosphate (DHAP) synthase, the first enzyme in the biosynthesis pathway of aromatic amino acids. As a consequence, phenylalanine overaccumulates in such mutants, causing enhanced flux also in its degradation pathway, resulting in higher 2-PE levels (64, 65). This fits with the observation that addition of high levels of l-phenylalanine in the medium also results in higher 2-PE levels (66). Naturally PFP-resistant mutants of *S. cerevisiae* accumulating high levels of 2-PE have not been reported.

The catabolism of l-phenylalanine in the Ehrlich pathway is initiated by the aromatic amino acid transferases encoded by *ARO8* and *ARO9* (67). There have been no reports of whether overexpression of *ARO8* and/or *ARO9* could enhance the level of 2-PE and thus also the level of its ester 2-PEAc. We have examined possible linkage in our QTL mapping results between the genomic position of *ARO8* (chromosome [Chr.] VII, 116059 to 117561) and *ARO9* (Chr. VIII, 375709 to 377250) and the high 2-PEAc production phenotype but did not find any indication for significant linkage in our QTL mapping. There was no significant difference between the BTC.1D and ER18 alleles regarding their contribution to the phenotype.

The first step in the amino acid catabolism to produce alcohols via the Ehrlich pathway involves the transamination into alpha-keto acid, catalyzed by *BAT1*, *BAT2*, *ARO8*, and *ARO9* genes (67). *BAT1* and *BAT2* are involved in the biosynthesis of branched-chain amino acids (valine, isoleucine, and leucine); however, overexpression of these genes could only poorly influence 2-PE levels, while isobutanol and isoamyl alcohol (and indirectly, isoamyl acetate) were significantly increased (68). However, overexpression of *ARO8* and *ARO9* was not evaluated. The first step in the conversion of phenylpyruvate to 2-PE is catalyzed by phenylpyruvate decarboxylase, which is encoded by *ARO10*, *PDC1*, *PDC5*, and *PDC6* (69). There was no linkage with any one of these genes in the QTL mapping of high 2-PEAc production. The same was true for the genes encoding phenylacetaldehyde dehydrogenase, *SFA1*, *ADH5*, and *ADH4*, catalyzing the second step in this conversion.

The next step in the production of acetate esters is the esterification of the higher alcohols, like 2-PE, with acetyl-CoA, which is catalyzed by the alcohol acetyl transferases *ATF1* and *ATF2* (4, 9, 11, 66, 70). This reaction allows the removal of 2-PE that can be cytotoxic at higher concentrations and production of the less harmful ester derivative 2-PEAc (66, 70). Ester formation can also regenerate the levels of free CoA in the cells (3, 71). Overexpression of *ATF1* led to a 2.4- to 10.8-fold increase in 2-PEAc production, depending on the substrate, yeast strain, and fermentation conditions. Overexpression of *ATF2* had only a minor effect, while *EHT1* overexpression had no effect (12). In spite of the important role of Atf1 in production of multiple flavor esters, including 2-PEAc, there was no linkage of high 2-PEAc production in our QTL mapping experiment with the genomic location of *ATF1* (Chr. XV, 1046226 to 1047803) nor with that of *ATF2* (Chr. VII, 848829 to 850436).

Adaptation of the flavor profile of alcoholic beverages by genetic modification poses multiple challenges. Genetic modification using deletion or overexpression of specific genes often produces unwanted side effects compromising industrial application of the engineered strains. Introduction of heterologous genes creates transgenic yeast strains, which currently are difficult to use for the production of alcoholic beverages and other foods. Exchange of naturally occurring *S. cerevisiae* alleles identified by polygenic analysis in existing natural or industrial yeast strains minimizes the risk of significant side effects on other cellular functions important for industrial application (18). It also creates cisgenic yeast strains, which may be or may become more acceptable for industrial application in the food sector (72–76). Since cisgenic yeast strains with precisely exchanged alleles cannot be recognized as GMOs, it is not possible in practice to apply GMO regulations to such organisms. In some countries, cisgenic or self-cloned organisms are not considered genetically modified organisms (60, 61, 77). Examples of self-cloned yeast strains that have received GRAS (generally recognized as safe) status are P1Y0 (GRN000350), which is a variant of a common *S. cerevisiae* wine strain with a superior *MET10* allele derived from another *S. cerevisiae* strain, and a baker’s yeast strain carrying extra copies of the natural *ASP3* gene (GRN000422).

### Conclusions.

We have identified two genes, the wild-type *TOR1* allele and a superior *FAS2*^BTC.1D^ allele, that affect the level of the important flavor compound 2-PEAc, which has a highly desirable rose flavor. The *FAS2*^BTC.1D^ allele allows us to strongly enhance the production of 2-PEAc, even in a strain that already produces a higher level than generally found in fermentations by *S. cerevisiae* strains. This is accomplished with additional positive effects on other flavor compounds and apparently without significant effects on compounds with a negative contribution to the flavor profile. Hence, the *FAS2*^BTC.1D^ allele appears to be highly promising for the creation of cisgenic brewing strains with an attractive and novel flavor profile.

## MATERIALS AND METHODS

### Microorganisms and cultivation media.

Yeasts were grown in yeast extract-peptone-dextrose (YPD) medium (2% [wt/vol] glucose, 2% [wt/vol] peptone, 1% [wt/vol] yeast extract) shaking at 200 rpm and at 30°C. For solid nutrient plates, 1.5% (wt/vol) Bacto agar was added. The *Saccharomyces cerevisiae* yeast strains used in this study are listed in Table 2.

**TABLE 2:** *S. cerevisiae* strains used in this study

Strain	Description^a^	Source and/or reference
WLP575	Industrial ale yeast strain	“White Labs Pitchable Liquid Yeast” (USA)
BTC.1D	Haploid segregant from WLP575; *MAT*α	This study
ER18	Segregant of Ethanol Red (industrial yeast strain used for bioethanol production); *MAT***a**	MCB, KU Leuven (21)
BTC.1D/ER18	Hybrid diploid strain obtained by crossing BTC.1D and ER18	This study
442	Haploid segregant from BTC.1D/ER18 with high 2-PEAc production	This study
442 *TOR1*Δ	Haploid 442 with *tor1*Δ (marker removed)	This study
442 *MOG1*Δ	Haploid 442 with *mog1*Δ (marker removed)	This study
442 *BNA2*Δ	Haploid 442 with *bna2*Δ (marker removed)	This study
442 *tor1*^E216^*	Haploid 442 with *tor1*^E216^* point mutation inserted via CRISPR/Cas9	This study
442 ChrXVI.b1	Haploid 442 with block 1 exchanged by ER18 allele (ChrXVI, 79486–85523)	This study
442 ChrXVI.b2	Haploid 442, block 2 exchanged by ER18 allele (ChrXVI, 85504–91015)	This study
442 ChrXVI.b5	Haploid 442, block 5 exchanged by ER18 allele (ChrXVI, 101093–104721)	This study
442 ChrXVI.b6	Haploid 442, block 6 exchanged by ER18 allele (ChrXVI, 104702–108350)	This study
442 ChrXVI.b7	Haploid 442, block 7 exchanged by ER18 allele (ChrXVI, 108331–114729)	This study
442 ChrXVI.b8	Haploid 442, block 8 exchanged by ER18 allele (ChrXVI, 114710–119641)	This study
442 ChrXVI.b9	Haploid 442, block 9 exchanged by ER18 allele (ChrXVI, 119622. 125450)	This study
442 ChrXVI.b10	Haploid 442, block 10 exchanged by ER18 allele (ChrXVI, 125432–129615)	This study
BTC2	Haploid BTC.1D with *tor1*^E216^*	This study
BTC3	Haploid ER18 with *TOR1*^E216^	This study
BTC4	Haploid BTC.1D with *fas*2^ER18^	This study
BTC5	Haploid ER18 with FAS2^BTC.1D^	This study
BTC6	Haploid ER18 with TOR1*^216E^/*FAS2*^BTC.1D^	This study

^a^ The chromosome (e.g., chromosome XVI) and positions for the block exchanged are shown within parentheses.

*Escherichia coli* cells (DH5; Invitrogen) were grown at 37°C in Luria broth (LB) medium containing 0.5% (wt/vol) yeast extract, 1% (wt/vol) Bacto tryptone, and 1% (wt/vol) sodium chloride (pH 7.5). For solid nutrient plates, 1.5% (wt/vol) Bacto agar was added. Selection of transformants was performed in the presence of 100 mg/ml ampicillin.

Flavor compound screening was performed in YP250Glu10% (0.27% yeast extract [Merck] and 0.54% Bacto peptone [Oxoid] to a total predicted nitrogen content of 250 mg/liter, which is in the same range as beer and wine fermentations, and 10% [wt/vol] glucose). The predicted nitrogen content was based on information of titratable nitrogen from the suppliers. For confirmation, unhopped wort was used (17°Plato, 18.8% [wt/vol] malt extract, 2.068 ppm ZnSO_4_ ⋅ 7H_2_O).

### Fermentation experiments.

Yeast cells were first cultivated overnight in 3 ml of YPD2% (yeast extract, peptone, 2% dextrose) in a shaking incubator at 30°C and 200 rpm. This preculture was used to inoculate 100 ml of YP250Glu10% (wt/vol) with an initial optical density at 600 nm (OD_600_) of 0.1. Fermentation was carried out at 25°C in 100-ml fermentation tubes, previously validated against European Brewing convention (EBC) tall tubes, fitted with a water lock in order to create semianaerobic conditions, mimicking large-scale fermentations. Agitation was performed with a magnetic rod at 130 rpm. Fermentation progress was monitored by weight loss due to CO_2_ release. Samples were taken at the end of fermentations for headspace gas chromatography coupled with flame ionization detection (GC-FID) analysis. Wort fermentations were performed at 20°C, and yeast was inoculated to an initial OD_600_ of 2.0.

### Headspace GC-FID analysis.

Headspace gas chromatography coupled with flame ionization detection was used to measure flavor compounds at the end of fermentation. Samples were collected and centrifuged at 3,500 rpm for 5 min. Then, 2-ml portions of the supernatant were collected in 25-ml vials and analyzed using a gas chromatograph with a headspace sampler (HS40; PerkinElmer Life Sciences). The headspace was equilibrated by shaking and incubating for 10 min at 60°C using a Thermoscience RS Plus auto sampler and then injected into a polyethylene glycol column (Restek Stabilwax) (60 m by 0.25 mm by 0.25 µm).

Injection block and flame ionization detector temperatures were kept constant at 220 and 250°C, respectively. The oven temperature was kept at 40°C for 2 min and then increased to 240°C at a rate of 15°C/min. Helium was used as the carrier gas at a flow rate of 2.0 ml/s. GC operating conditions were as follows: injection volume of 1 ml, split rate of 1:25, and split flow of 50 ml/min.

### Mating type, sporulation, and tetrad dissection.

Standard procedures were used for sporulation and tetrad dissection (78) and for mating type determination by PCR with primers for the *MAT* locus and *MAT***a** and *MAT*α DNA (79).

### Molecular biology methods.

Yeast cells were transformed by electroporation (80). Standard molecular biology protocols were used in this work.

### Genomic DNA extraction and whole-genome sequence analysis.

Segregants were grown separately in 3 ml YPD and pooled by OD_600_ with the purpose of obtaining approximately equal amounts of DNA for each strain. Genomic DNA was extracted and purified with the Masterpure Yeast DNA purification kit from Epicentre in order to obtain high-quality genomic DNA. At least 10 µg of DNA per pool was provided to the Beijing Genomics Institute (BGI) (Hong Kong, China) for whole-genome sequence analysis, which was performed with the Illumina platform (HiSeq2000).

Assembly and mapping were performed with NGSEP (next-generation sequencing eclipse plugin) (81). Significance tests used to determine quantitative trait loci (QTLs) were conducted by the method of Claesen et al. (82).

### Next-generation sequence analysis and statistical analysis.

The sequence reads from the parental strains and the pool were first aligned to the reference S288c genome sequence after which single nucleotide polymorphisms (SNPs) remaining between the two parent strains and with a coverage of at least 20 times were selected following previous polygenic analysis protocols (17, 38). SNPs between the individual parent strains and the reference genome sequence but absent in the parent strains themselves were filtered out using the next-generation sequencing eclipse plugin (81). SNP variant frequencies were calculated by dividing the number of the variant by the total number of aligned reads. Deviation from 50%, either above or below, was a sign of a one-sided SNP variant segregation, indicating genetic linkage to the trait of interest (18).

Genetic linkage to the phenotype of high phenylethyl acetate (2-PEAc) production was statistically confirmed using EXPloRA, a hidden Markov model (HMM)-based algorithm for extraction of overrepresented alleles in bulk segregant analysis (83). Finally, the SNP variant frequency mapping and data smoothing were performed using the HMM-based algorithm developed by Claesen and Burzykowski (84) in the R platform (RStudio 0.99.903 program, R version 3.3.1, R Development Core Team, 2013). This algorithm allowed reduction of the width of QTLs by estimating the significance (*P* value) of linkage in each SNP location by comparing the SNP variant frequencies of the superior and random pools at this position. In addition, we have calculated the *P* value by comparing the SNP variant frequency of the superior pool and a predicted variant frequency of a random pool of 0.5, which would correspond, in the absence of inadvertent linkage, to the expected situation that for the total number of reads at a certain position, half represent the reference SNP variant and the other half represent the alternative SNP variant.

### Allele-specific PCR.

Fine mapping was performed by scoring selected SNPs in the 24 individual superior segregants. SNPs were chosen throughout each QTL at an average distance of 5 to 15 kb. For each SNP marker, two primers were constructed differing only at their 3′-terminal end, containing either the BTC.1D or ER18 nucleotide. To select these primers, the same criteria were taken into consideration as for any regular primer. Moreover, the SNP located at the 3′-terminal end should ideally vary from A or T to C or G and vice versa to improve the chances of specifically amplifying the BTC.1D orER18 sequence.

Both primers were always applied in separate PCRs. The optimal annealing temperature was determined by gradient PCR using DNA from BTC.1D and ER18 parents. The optimal temperature is the annealing temperature at which only hybridization with primers containing an exact match was observed. The SNP data of the individual segregants were analyzed using the binomial distribution probability.

### CRISPR/Cas9 technology. (i) Cas9 plasmid.

The clustered regularly interspaced short palindromic repeat (CRISPR)/Cas9 technology was applied by using the pTEF-Cas9-KanMX plasmid (CRISPR-mediated gene inactivation or replacement, single copy, Cas9 behind the TEF promoter, KanMX marker) which was derived from the p414-TEF1p-Cas9-CYC1t plasmid (27). The pTEF-Cas9-KanMX plasmid was created by introducing the KanMX4 marker into KpnI-digested p414-TEF1p-Cas9-CYC1t.

### (ii) gRNA plasmids.

For each target, specific guide RNAs (gRNAs) were designed for 1x-, 2x-, or 3xgRNA approach. Specific gRNAs were assembled in the XhoI-EcoRV-digested P58 vector by using the Gibson Assembly kit. P58 was derived from p426-SNR52p-gRNA.CAN1.Y-SUP4t (27). It was created by assembling the HPH marker and the universal CRISPR gRNA cloning site into the p426-SNR52p-gRNA.CAN1.Y-SUP4t backbone.

*(a) 1xgRNA approach—introduction of point mutation.* A specific gRNA without a protospacer adjacent motif (PAM) site and flanked by flanking regions 1 and 2 was designed. Flanking region 1 (promoter) was GCAGTGAAAGATAAATGATC. Flanking region 2 (terminator) was GTTTTAGAGCTAGAAATAG.

Forward and reverse oligomers were hybridized and assembled in the XhoI-EcoRV-digested P58 vector with the Gibson Assembly kit. For hybridization, oligomers were dissolved in STE buffer (10 mM Tris [pH 8.0], 50 mM NaCl, 1 mM EDTA) at a concentration of 500 µM. Equimolar concentrations of the forward and reverse primers, were combined and heated at 94°C (4°C/min) for 3 min and slowly cooled down (to 75°C at 0.05°C/min and then cooled to 10°C at 0.02°C/min).

*(b) pJET-2xgRNA.* The pJET-2xgRNA plasmid was derived from pJET1,2-blunt (ThermoScientific) and contains a module consisting of the gRNA terminator and promoter. Amplification of this module, flanked by gRNA sequences, allows the addition of more gRNAs in the regular gRNA plasmids.

*(c) 2xgRNA approach—allele swapping in haploids.* A specific gRNA without a PAM site and flanked by flanking regions 1 and 2 was designed, one before the first SNP of interest and the other after the last SNP of interest. Forward gRNA1 and reverse gRNA2 oligomers were used to amplify the module from pJET-2xgRNA. Amplification of this module flanked with gRNA sequences allows the addition of more gRNAs in the regular gRNA plasmids. The amplification product (505 bp) was assembled in the XhoI-EcoRV-digested P58 vector using the Gibson Assembly kit.

### Design guide RNA targets.

Proper gRNA targets were selected based on the presence of natural SNPs in the inferior and superior alleles at the protospacer adjacent motif sequence or the 8 bp of DNA preceding the PAM sequence, reported to be the most important for specificity (85, 86). Next, we analyzed potential off-targets in the genome, which are regions matching the gRNA sequence with three or less SNPs followed by a valid PAM site.

### Donor DNA. (i) Introducing a point mutation in TOR1.

Donor DNA was a 71-bp oligomer containing the desired mutation and 35-bp identical sequences on each side: TAAAGTCTTGCCTAGAATGGCTTACTGCCTCCACG**T**AAAAGAATTCATTCTCAAGTTCGAAGCCAGACCAT.

The forward and reverse sequences were ordered and then hybridized.

### (ii) Allele swapping.

Donor DNA was PCR amplified from the BTC.1D parent, which contains the superior allele on chromosome XVI. Donor DNA contained 500 to 800 bp of flanking sequence similarity.

### CRISPR/Cas9 application.

Point mutations were introduced by using CRISPR/Cas9 technology and a single gRNA. Bulk replacements on chromosome XVI were performed by using the 2xgRNA approach. In haploid cells, the 1x- or 2xgRNA approach was used. Cells were first transformed with 250 ng of pTEF-Cas9-KanMX plasmid by electroporation. Cas9 transformants were pregrown overnight in 3 ml YPD-Geneticin (200 µg/ml) at 30°C. This culture was used to inoculate 50 ml YPD-Geneticin to an initial OD of 0.2. Cells were transformed by electroporation using 500 ng of the gRNA plasmid and 1,000 ng of donor DNA (or 2 μl of 250 μM duplexed oligomers for the point mutation). Transformants were selected on solid YPD2% plus 200 µg/ml Geneticin and 300 µg/ml hygromycin B.

### Data availability.

All sequence data have been submitted to the NCBI Sequence Read Archive (SRA) (http://trace.ncbi.nlm.nih.gov/Traces/sra/sra.cgi) with the identifier SRP119133, Bioproject PRJNA412158.
